# Bone metabolism in primary hyperparathyroidism

**DOI:** 10.1007/s00508-025-02670-z

**Published:** 2025-12-02

**Authors:** Katharina Kerschan-Schindl, Katharina Gelles, Maria Butylina, Richard Crevenna, Peter Pietschmann

**Affiliations:** 1 Department of Physical Medicine, Rehabilitation and Occupational Medicine, Medical University of Vienna, Vienna, Austria; 2 Comprehensive Center of Musculoskeletal Disorders (CCMSD), Medical University of Vienna, Vienna, Austria; 3 Institute of Pathophysiology and Allergy Research, Center for Pathophysiology, Infectiology and Immunology, Medical University of Vienna, Vienna, Austria

**Keywords:** Bone turnover marker, Bone mineral density, Fracture risk, Therapy, Osteitis fibrisa cystica

## Abstract

Primary hyperparathyroidism is a frequent endocrine disorder that affects various organ systems. In this review we present and discuss alterations of bone metabolism in primary hyperparathyroidism. Excessive secretion of parathyroid hormone results in increased bone remodelling with an excess of bone resorption. Consequently, bone mineral density declines, bone quality is compromised and fracture risk increases.

Successful surgery for hyperparathyroidism results in a normalization of bone turnover and a decrease of fracture risk. Osteitis fibrosa cystica, a severe bone manifestation of hyperparathyroidism, is observed rarely today.

## Introduction

Under physiological conditions parathyroid hormone (PTH) consisting of 84 amino acids, ensures maintenance of normocalcemia sustaining the ionized calcium levels (Ca2+) within a narrow range. If the calcium sensing receptor located in the parathyroid cells detects hypocalcemia, the parathyroid gland releases PTH. Bone, kidneys and gut are the target organs of this polypeptide. As approximately 99% of the total calcium is located in bone, bone is the major reservoir for repletion of serum calcium deficits. The calciotropic action of PTH under physiological conditions is a self-limiting feedback process, whereas autonomous production of PTH caused by primary hyperparathyroidism (PHPT) is associated with an ongoing impact of bone turnover.

The aim of this manuscript is to describe changes in bone metabolism and bone strength accompanying PHPT. Additionally, it addresses the positive effects on regeneration of bone metabolism treating the disease.

## Molecular pathways of parathyroid hormone

The PTH activates different molecular pathways based on the activation of the PTH 1 receptor (PTH1R), a G protein-coupled receptor, which is expressed on various bone cells. Intermittent PTH binding induces anabolic effects. Continuously elevated PTH levels produce a higher number of PTH1R, thus leading to a preponderance of catabolic actions. Continuous stimulation of osteoclasts by the PTH1R induces an increase in the resorption activity [1, 2]. The binding of PTH on the PTH1R of the osteoblasts starts a complex cascade of intracellular signalling, an increase in cyclic adenosine monophosphate (CAMP) production, followed by activation of protein kinase A (PKA), which besides others phosphorylates CAMP response element binding protein (CREB), a transcription factor increasing receptor activator nuclear factor-кB ligand (RANKL) and decreasing osteoprotegerin (OPG) production (for review see [3]). The RANKL/OPG system plays an important role in bone metabolism. In PHPT, RANKL is assumed to be elevated [4–6] and OPG seems to be not affected or decreased [4, 6, 7]. The binding of PTH to PTH1R on osteocytes induces the inhibition of salt-inducible kinase 2 (SIK2) by activated PKA. As a result, sclerostin (SOST) expression is suppressed [8].

The Wnt pathway influences the bone formation through effects on osteoblast function and these actions are opposed by SOST and dickkopf‑1 (DKK1).

In PHPT patients serum SOST levels seem to be reduced [9–12]. Besides the decreased SOST levels Viapiana et al. [12] also detected elevated levels of the protein DKK1 in PHPT patients compared with a control group. No further studies investigating DKK 1 in PHPT patients exist. Elevated PTH levels are also associated with proinflammatory markers [13]. Besides bone cells, T lymphocytes also express PTH1R. In a study by Tawfeek et al. [14] it was demonstrated that continuous PTH elevation stimulates T lymphocytic tumor necrosis factor (TNF)-alpha production, which leads to synthetization of interleukin (IL)17‑A. In PHPT patients IL17‑A is upregulated and IL17‑A inhibitors are able to prevent bone loss [15]. The resorptive enzyme cathepsin K was investigated only once and was not elevated in patients with PHPT [32]; however, a cross-sectional study investigating 25 fractured and 25 nonfractured postmenopausal women with PHPT detected higher serum levels of k‑periostin, a degradation product of cathepsin K, in fractured patients [16]. Fibroblast growth factor (FGF) 23, a regulator of the calcium-phosphate metabolism, was evaluated in PHPT patients and shown to be either normal or elevated [17–20] with positive correlations between PTH and FGF23 [18].

To sum up, PTH binds to its receptor, which is expressed on the surface of several bone cells, and thus initiates different molecular pathways influencing bone metabolism.

## Bone turnover in PHPT

**Fig. 1:**
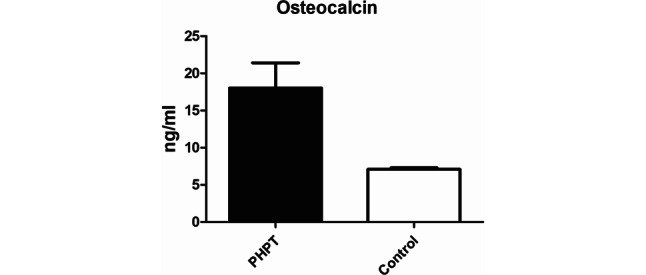
Serum osteocalcin levels in patients with primary hyperparathyroidism (*PHPT*) and 24 controls. Data are means ± SEM and were obtained from Pietschmann et al. [34]

Bone turnover markers (BTM) reflect the effect of PHPT on bone. Only a few investigations showed BTMs in the (upper) normal range [21–23]. Most studies detected elevations of bone resorption markers, including C‑terminal telopeptide of type I collagen (CTX), N‑terminal telopeptide of type I collagen (NTX), NTX/creatinine, urinary NTX/creatinine, urinary pyridinoline, urinary pyridinoline/creatinine, urinary hydroxyproline/creatine, urinary deoxypyridinoline/creatinine, urinary cross-linked N‑telopeptides of type I collagen/creatinine urinary galactosyl hydroxylysine/creatinine [4, 24–32] and bone formation markers, which included osteocalcin (Fig. 1), alkaline phosphatase, bone-specific alkaline phosphatase, procollagen type I C‑terminal propeptide (PICP) and procollagen type I N‑terminal propeptide (PINP) [26–38]. The general bone turnover marker osteocalcin (a bone protein produced by osteoblasts regulating mineralization) but especially bone resorption markers (measuring the rate of bone breakdown by tracking collagen degradation products released by osteoclasts) are assumed to be elevated in PHPT patients with skeletal involvement [39]; however, as the value of BTM to predict the extent of skeletal involvement or amelioration of bone metabolism after surgery is uncertain, their evaluation is not generally recommended [40].

## Quantitative aspects of bone in PHPT—Bone mineral density

The inability of osteoblasts to compensate the action of osteoclasts leads to a net increase of bone resorption. A prospective study evaluating BMD of 140 patients with PHPT, detected osteoporosis in 63% of the patients [41] and it is more often present in women than in men [42]. Even in a mild form of PHPT, BMD was reduced [43]. An observational study (up to 15 years) showed that BMD of the lumbar spine remained relatively stable whereas BMD at cortical sites decreased (10% at the femoral neck and 35% at the distal radius) [44]; however, in postmenopausal women, postmenopausal osteoporosis may potentially mask typical PHPT-associated predominance of cortical bone loss. A reduced BMD can be present at several measured regions, those with a high percentage of cortical bone (distal radius), those with a more mixed bone composition (hip), and those with a high amount of cancellous bone (lumbar spine) [45]. Thus, BMD should not only be measured at the lumbar spine and hip region but at the distal 1/3 radius as well; a reduction of BMD to a T score ≤ 2.5 at any site is one of the criteria for recommending surgery [40].

## Qualitative aspects of bone in PHPT—Bone quality

The trabecular bone score (TBS) is a standard parameter of the lumbar spine. Measurement of BMD using dual energy absorption gives information on the microarchitecture and, thus, provides an indirect measure of bone strength. Mean TBS values have been shown to be significantly reduced in PHPT compared to controls [46–48]. According to a retrospective analysis [49] TBS is reduced in PHPT patients; however, after adjusting for the BMD of the lumbar spine, which was also decreased in patients compared with matched controls, the difference in the TBS score was no longer significant [49]. High-resolution peripheral quantitative computer tomography (HR-pQCT) had been used a few times to assess bone geometry in PHPT patients. In contrast to one study, which only detected microarchitectural deteriorations in the radius [50], Stein et al. [51] found microstructural abnormalities (decreased cortical and trabecular volumetric densities, reduced cortical thickness and a larger trabecular distribution) in the tibia as well. A Chinese investigation confirmed the deficits of the trabecular and cortical bone compartments in female and male PHPT patients [52]. Histological data from iliac crest biopsies revealed a higher osteoid surface and osteoid seam thickness (because of high bone turnover) of 11 PHPT patients compared to controls [53]. Quantitative backscattered electron imaging of iliac crest bone biopsies showed a reduction of the degree of mineralization accompanied with an increase in the heterogeneity of the amount of mineralization [54]. The accelerated bone turnover may lead to these histological changes.

## Risk of fragility fractures

Reduction of BMD and degradation of bone microarchitecture indicate a reduced bone quality leading to susceptibility to fragility fractures. Indeed, two meta-analyses including 12 and 17 studies, respectively, confirmed an increased risk of fractures [55, 56]. Narayanan et al. [56] found an increased risk of total fractures (relative risk, RR 1.71) and vertebral fractures (RR 2.57) but no significant difference concerning nonvertebral fractures (RR 1.37). Ejlsmark-Svensson et al. [55] detected an increased risk of any fracture (odds ratio, OR 2.01); their analyses of different fracture sites revealed a significantly increased risk at the spine (OR 3.00) and forearm (OR 2.36) as well as a nonsignificant increase in the risk at the hip region (OR 1.27). According to a more recent Swedish nationwide cohort study [57] PHPT patients have an increased incidence rate of any fracture (IRR 1.27).

## Osteitis fibrosa cystica

Osteitis fibrosa cystica (OFC) documents a severe bone manifestation and is characteristic for hyperparathyroidism [58]. It was first described in 1891 by Recklinghausen in a Festschrift dedicated to Virchow [59]. The OFC is not specific for PHPT as it is also observed in the secondary and tertiary variants of the disease [60] and results from the osteolytic overactivity of PTH [61]. The lesions can be unifocal or multifocal. Histologically the lesions contain multinucleated giant cells, fibrous tissue, hemorrhages and hemosiderin deposition; the latter results in a brownish coloration of the tissue [60, 62]. Therefore, the lesions are also referred to as “brown tumors” (although they are not to be considered as neoblastic). Brown tumors can be present at many skeletal sites including the jaws, pelvis and long bones; X‑ray findings include lytic or cystic lesions [62] (after successful surgery for PHPT OFC usually regresses, nevertheless, in some lesions additional orthopedic surgery may be necessary [60]).

In the first half of the twentieth century OFC was a typical presentation of PHPT. Due to the availability of automated methods for blood calcium determinations, diagnosis of PHPT is made earlier, thus OFC is seen less frequently [63]. In 2021, Arya et al. [64] reported that 28% of premenopausal Indian women with PHPT presented with OFC (in postmenopausal women the incidence was 13%). A study from Brazil reported a prevalence of 6% [65] whereas in a case series from Italy OFC was present only in 0.5% of patients who had surgery for PHPT [66]. In clinical practice, OFC can be misdiagnosed as bone malignancy or bone metastases [60, 62]. Therefore, the knowledge of this now rare disease manifestation is still important to avoid misdiagnosis.

## Treatment

According to the European expert consensus on the management of PHPT, patients with PHPT usually do not need to restrict their calcium intake but should not exceed the recommended daily calcium intake [67]. In PHPT patients with vitamin D deficiency, vitamin D supplementation was proven to reduce PTH levels without causing hypercalciuria or elevated serum calcium levels [68].

### Effect of surgery on bone disease

Parathyroid surgery (PTX) is the treatment option, which definitively cures the disease. An intraoperative reduction of PTH levels by half within 10 minutes after adenoma resection indicates removement of all hyperfunctioning parathyroid tissue [69]. The day after surgery, normal total and ionized as well as PTH levels predict cure [32]. According to a meta-analysis including 6 trials (441 patients), surgery achieves long-term biochemical cure in 96.1% [70]. Bone resorption markers decrease within a few hours after surgery; however, the reduction of the bone formation markers lags behind [29]. Serial analyses of the BTM of PHPT patients (classically symptomatic, minimal symptomatic or asymptomatic) revealed a steady decrease of osteocalcin and bone-specific alkaline phosphatase the first year after surgery, whereas the bone resorption marker C‑terminal telopeptide of type I collagen (CTX) showed a different pattern. A transient drop within the first 24 h after surgery was followed by a slight increase and thereafter by a slow and steady decrease within the year after PTX [32]. A retrospective investigation of BTM 6–12 months after PTX also showed significantly lower serum levels of bone formation (procollagen type 1 N‑terminal propeptide and bone-specific alkaline phosphatase) and bone resorption marker CTX (C-terminal telopeptide of type 1 collagen or C‑telopeptide cross-linked type I collagen) compared with presurgery values [71]. Greater declines of BTM after PTX are assumed to be associated with higher increases of BMD after surgery [22]. A prospective study showed that BMD of the lumbar spine increased significantly within 1 year whereas BMD of the femoral neck did not change significantly [32]. Guo et al. [26] detected significant bone gains at total body, lumbar spine and femoral neck 2 years after surgery. A meta-analysis of 4 trials investigating BMD changes up to 5 years after surgery revealed an increase of lumbar spine BMD (MD 4.82), total hip BMD (MD 4.41) and femoral neck BMD (MD 3.18), although the latter was not significant [70]. Compared to not surgically treated patients, PTX patients have higher annual percentage BMD gains at the lumbar spine, femoral neck, total hip and radius [72]. Additionally, early surgical intervention seems to be associated with higher BMD increases than a larger intervention time window [73].

The increased fracture rate in PHPT patients, which is highest the last year before surgery, normalizes thereafter [57]. A retrospective study of 60 PHPT patients showed that the 10-year risk of major osteoporotic (MOF) fractures and hip fractures determined by the FRAX tool (https://frax.shef.ac.uk/frax/) remains stable after PTX, whereas it increases in PHPT patients without surgery [74]. Compared to observation, PTX significantly reduces fracture risk (RR 0.80); looking at the different regions, fracture risk reduction was not significant for the forearm and vertebral bodies but significant for the hip region (RR 0.63) [72].

A Swedish study group showed that calcium plus vitamin D supplementation following parathyroidectomy led to a lower serum level of PTH besides the increased serum levels of 25-OH-vitamin D compared to a group only receiving calcium supplementation [75]; however, BTM and BMD did not differ significantly between the study groups [76].

The frequency of calcium monitoring depends on the serum levels the day after surgery and on the clinical situation [77]. Concerning the evaluation of BTM after successful surgery, no guidelines exist. In patients with low BMD before surgery, a reassessment of BMD the year after surgery is recommended [77].

### Effect of pharmacological treatment on bone disease

In the case of patients with severe comorbidities, the risk of surgery may outweigh its benefits. Additionally, some patients may refuse surgery. According to the guidelines for the management of PHPT-patients [40] these patients should receive the calcimimetic agent cinacalcet in order to reduce the serum calcium level, if necessary, calcium and vitamin D supplementation and a bone-specific treatment. Antiresorptive drugs proved to have positive effects on BMD in PHPT patients [78, 79]. Patients who do not undergo surgery have to be monitored regularly. According to Bilezikian et al. [40], serum levels of calcium and vitamin D as well as the estimation of renal function (creatinine clearance or estimated glomerular filtration rate) should be evaluated at least once a year and BMD measurement every year or every other year. Nevertheless, in the SFE-AFC-SFMN 2024 consensus on primary hyperparathyroidism [77] it is recommended to determine serum calcium levels every 3 months in PHPT patients on medical treatment.

In patients undergoing PTX, BMD increases as described above; however, bone gain is affected by osteoporosis treatment. According to a retrospective study, PTX in bisphosphonate-naïve patients and PTX following bisphosphonate treatment leads to a lower risk of fragility fractures within 2 years following surgery compared to a bisphosphonate start after PTX [80]. For this reason, it has been suggested to start antiresorptive treatment 2 years after successful surgery [81]; however, Ryhänen et al. [82] showed that a start of zoledronate treatment as early as 1–3 months after PTX increased BMD to a significantly higher extent than no antiresorptive treatment the first 2 years after surgery. The daily intake of strontium ranelate, calcium and vitamin D initiated 4 weeks after PTX led to a higher BMD increase the year following surgery compared to the control group (supplementation with calcium and vitamin D) [83]. Bone resorption as well as bone formation markers drop after surgery. Thus, dual mode of action preparations (which increase bone formation) may be an interesting option in PHPT patients after PTX.

## Conclusion

Although the clinical presentation of PHPT has significantly changed over time, bone manifestations are still of clinical relevance. According to the current evaluation and management guidelines [40], three-site DXA measurement (lumbar spine, hip, distal radius) and X‑ray or vertebral fracture assessment (VFA) are essential methods to determine PHPT associated bone disease; if available, the TBS is helpful in determining skeletal involvement. Biochemical evaluation of bone turnover is not generally recommended [40]. The surgical cure of the disease leads to a normalization of bone turnover. In contrast, the pharmacological management of patients after successful surgery requires further study.
